# Family Planning, Reproductive Health, and Progress Toward the Sustainable Development Goals: Reflections and Directions on the 30th Anniversary of the International Conference on Population and Development

**DOI:** 10.9745/GHSP-D-24-00127

**Published:** 2024-10-29

**Authors:** Shyami de Silva, Apoorva Jadhav, Madeleine Short Fabic, Loyce Munthali, Foyeke Oyedokun-Adebagbo, Zewditu Kebede

**Affiliations:** a U.S. Agency for International Development, Washington, DC, USA.; b U.S. Agency for International Development, Lusaka, Zambia.; c U.S. Agency for International Development, Abuja, Nigeria.; d U.S. Agency for International Development, Addis Ababa, Ethiopia.

## Abstract

Investing in family planning and reproductive health—broadly defined as the services, policies, information, attitudes, practices, and commodities, including contraceptives, that help individuals achieve their fertility intentions—is integral to attaining many Sustainable Development Goals.

## Key Messages

- On the 30th anniversary of the watershed International Conference on Population and Development, we reflect on recent influential shifts impacting family planning (FP) and the growing evidence base on the importance of investing in FP.
- We describe the U.S. Agency for International Development’s (USAID) Pathways to Progress for Sexual and Reproductive Health, 2024–2030, a forward-leaning framework that guides USAID’s family planning and reproductive health programs to help realize a world where ongoing improvements to sexual and reproductive health (SRH) contribute to longer, healthier, and more prosperous lives for all.
- We also delineate the 3 evidence-based pathways to achieve that vision that operate at individual, systems, and societal levels:
  - Individuals have accurate information, skills, and ability to take action to achieve the highest attainable levels of SRH across their lifetime.
  - Health systems provide quality, accessible, and people-centered SRH care.
  - Local communities, organizations, institutions, and governments create and foster social norms and policies that support individuals to make and act on their own SRH decisions, free from violence, coercion, and discrimination.

## WHY NOW?

The landmark 1994 International Conference on Population and Development (ICPD) shifted the dominant rationale for family planning (FP) from a demographic context that centered concerns about rapid population growth to a human rights context that centered people’s dignity and rights.^1^ The ICPD Programme of Action has enduring relevance. On the 30th anniversary of ICPD (ICPD+30), the global community of FP stakeholders is still striving to meet individuals’ sexual and reproductive health (SRH) needs while doing so using person-centered, rights-based approaches and measures of progress.

As members of the global FP community who work at the world’s largest bilateral donor in FP, the U.S. Agency for International Development (USAID), we are taking the opportunity of ICPD+30 to consider what we have learned and where the field is headed. This commentary offers a synthesis of our reflections, beginning with a summary of recent influential shifts impacting FP, followed by a summary of the growing evidence base on the importance of investing in FP, and concluding with a description of USAID’s vision for the future—a world where ongoing improvements to SRH contribute to longer, healthier, and more prosperous lives for all. To be clear on the scope of USAID’s support for SRH within the “FP/reproductive health (RH)” funding earmark in the annual appropriations act, in addition to comprehensive, voluntary FP, USAID also implements activities that address FP integration with HIV/AIDS and maternal health (such as postabortion care); child, early, and forced marriage; gender-based violence (GBV); female genital mutilation/cutting; and fistula. All program-supported FP services are guided by the principles of voluntarism and informed choice. USAID does not fund abortions.

## RECENT INFLUENTIAL SHIFTS IMPACTING FAMILY PLANNING: REASONS FOR OPTIMISM AND PESSIMISM

Since ICPD, the world has taken the opportunity every 5 years to assess progress toward the Programme of Action. The 25th anniversary of ICPD was greeted with a collection of commentaries assessing progress^2^^,^^3^ with some offering a “glass half full” assessment recognizing major advances, including significant impacts on national laws and policymaking, program development and service delivery, and others offering a “glass half empty” assessment, recognizing that many individuals and communities are still left behind and many key SRH issues are still un- or under-addressed.^4^ The most recent 5-year period leading to ICPD+30 has similarly been met with opportunities and challenges, giving credence to the perspectives of optimists and pessimists alike. On the side of the optimists, we have the recent reassessments of FP goals and measures alongside growing global commitments to advancing individual rights and reproductive agency. On the side of the pessimists, we have flatlined budgets alongside compounding global crises.

### Shifting Perspectives and Measures of “Success”

In the most recent 5-year period, scholars and practitioners alike have called for a reassessment of how FP programs define and measure success: moving beyond established measures of success (like contraceptive use) toward more person-centered and/or human rights-based measures that focus on agency, equity, and empowerment to truly reflect and embody the fundamental tenets of the ICPD Programme of Action.^5^^,^^6^ Concurrently, the global FP community is grappling with the implications of this shift; how complex concepts, such as agency and empowerment, are defined, measured, and advanced; and the degree to which FP programming must evolve to better meet an individual’s SRH needs. This work is transpiring in the context of global megatrends, including climate change, conflict, forced migration, pandemics, and other stressors, which alone and in combination present threats to individual and community health.

Scholars and practitioners have called for a reassessment of how FP programs define and measure success toward more person-centered and/or human rights-based measures that focus on agency, equity, and empowerment.

This reassessment was well captured in a special 2023 issue of *Studies in Family Planning* journal that presented a collection of studies that critiqued existing program-centered indicators and offered new ones reflective of person-centered, rights-based goals and programming.^7^ Similarly, *GHSP* has also recently published articles on this topic, breathing in fresh perspective for the FP field.^8^^,^^9^

USAID is doing its part to advance this person-centered, rights-based FP measurement work, including through its more than 40 years of leadership of the Demographic and Health Surveys (DHS) Program—which has and will continue to evolve to address the most pressing issues facing women and girls, including new measures of “success”—as well as through new avenues of investment, such as USAID’s Agency for All project.^10^ This project works with local partners to generate and apply evidence on the role of agency—an important piece of the measurement agenda in SRH—in effective social and behavior change programming to improve the health and well-being of individuals and communities.

While a full summary of measurement advancements is beyond the scope of this commentary, the critical thinking, research, and expanded evidence base presented in this work represent an actualization of the human rights-affirming commitments of ICPD.^8^^,^^9^^,^^11^^,^^12^

### Amplifying Rights and Agency Through Global Commitments and Partnerships

Global commitments and goals are shifting. Take, for example, FP2030, which is the next iteration of FP2020, borne out of the 2012 London Summit.^13^ FP2030 has the explicit aim of “forging a path to a future where everyone, everywhere, has the freedom and ability to make their own informed decisions about using modern contraception and whether or when to have children, to lead healthy lives, and to participate as equals in society and its development.”^14^ While FP2030 outlines their measurement agenda, including an iterative approach to incorporating new measures based on intentional local stakeholder engagement,^15^ this focus on individual agency is in contrast to the numeric goal set forth by FP2020, “to enable 120 million additional women and girls to use contraceptives by 2020,”^13^ which was critiqued by many advocates and practitioners as a regressive step away from ICPD.^16^^,^^17^ Other key groups supporting SRH, including the Ouagadougou Partnership and the United Nations Population Fund (UNFPA), have also made important efforts to more fully embrace the ICPD agenda. For example, the Ouagadougou Partnership, established in 2011 by 9 West African countries with an initial focus on increasing contraceptive use among youth,^18^ has now expanded to include innovative strategies on multidimensional research, social norms, and FP in humanitarian contexts, with increased input from civil society.^19^ Similarly, within the last 5 years, the UNFPA has more clearly articulated its commitment to “increased focus on the rights and inclusive participation of women, adolescents and youth as agents of change” while “leaving no room for discrimination or exclusion,” as elaborated in UNFPA’s Strategic Plan (2022–2025).^20^ USAID, too, has actively worked to amplify the ICPD agenda and elevate rights-based FP, most recently articulating its approach in *Pathways to Progress for Sexual and Reproductive Health*, *2024–2030* (further described herein). Furthermore, with USAID’s refreshed approach to localization^21^—with the intention of local actors leading and driving the USAID’s work—comes the opportunity for a shift in power to marginalized groups, which dovetails with the ICPD Programme of Action’s focus on addressing inequalities and inclusive development.

### Stagnating Budgets in the Face of Growing Populations of Reproductive Age and Polycrises

These innovations in thinking, measurement, and approach are much needed and are increasingly responsive to the ICPD Programme of Action. They are also occurring in the context of flatline funding for FP. In 2022, donor governments provided US$1.35 billion in bilateral funding for FP activities (with notable variations by country), an amount essentially unchanged from 2020.^22^ Meanwhile, the other 2 major sources of FP funding—domestic government spending and out-of-pocket expenditures—were also essentially flatlined; total FP expenditures were estimated to be 1% lower in 2021 compared to 2020.^23^ As the global FP community aims to meet the SRH needs of growing cohorts of individuals of reproductive age, while identifying and acting on opportunities to better address access, equity, and choice, this flatline budget represents a major obstacle.^24^

Innovations in thinking, measurement, and approach are much needed and increasingly responsive to the ICPD Programme of Action while occurring in the context of flatline funding for FP.

Complicating matters further are the compounding crises of today, highlighting a need for preparedness, coordination, and community engagement. These challenges include climate, conflict, and global health insecurity, which negatively affect quality of life and well-being globally, but, in particular, the mental health and SRH of women and adolescents.^25^^–^^28^

## GROWING EVIDENCE BASE ON THE IMPORTANCE OF INVESTING IN FAMILY PLANNING AND GLOBAL PROGRESS TOWARD ACHIEVING THE SUSTAINABLE DEVELOPMENT GOALS

In 2016, a group of authors from USAID described investments in FP as a development “best buy” with the potential to accelerate achievement across the core Sustainable Development Goal (SDG) themes of people, planet, prosperity, peace, and partnership.^29^ Since then, a wealth of new research and scientific evidence has become available, much of which bolsters the framing set in that 2016 article, though with some key nuances. We share the following key findings of recent research.

### People

The core SDG subgrouping of “people’’ includes poverty, nutrition, maternal mortality, education, and empowerment. Most research relating FP to this subgrouping has used cross-sectional surveys to examine relationships (i.e., correlational rather than causal). In recent studies, researchers have concluded that contraceptive use is associated with increased urbanization and reduced poverty.^30^^–^^32^ Other researchers have found strong relationships between contraceptive use and improved nutritional status of women and children^33^^,^^34^ and food security.^35^ Importantly, researchers have identified FP as a key component of food and nutrition interventions that improve socioeconomic status and parental nutritional status.^36^ Turning to maternal mortality, researchers have modeled the impact of FP on maternal survival and other maternal health outcomes, results from which have supported the scale-up of FP services in Ethiopia,^37^ Indonesia,^38^ and South Africa.^39^ Other researchers have found that integrating HIV and FP services can be an efficient way to expand service utilization and reduce maternal and child morbidity and mortality.^40^^,^^41^

The FP field is crowded when considering women’s empowerment in the “people’’ SDG category. Many studies have analyzed the complex bidirectional relationship between women’s empowerment and FP across different country and cultural contexts, such as recent studies from Ethiopia,^42^ Burkina Faso,^43^ and Mozambique.^44^ Others have analyzed the relationship between reproductive empowerment and FP self-care.^45^^,^^46^ Others still have offered nuanced differentiation between the exercise of choice and the existence of choice.^47^ The succinct conclusion offered by recent proceedings from a related National Academies deliberation is this: FP is necessary but not sufficient for women’s empowerment.^48^ FP is an essential component of many aspects of sustainable development. Indeed, FP has been identified as a “best buy” for Africa, along with vaccinations for rotavirus, expanded malaria interventions, and preschool education, among others.^49^

### Planet

Of the SDGs, FP’s connection with the “planet” component can be the most contentious if the focus is on population growth. Recently, for example, a leading demographer made the argument that population growth is a key driver of climate change, greater and more effective use of contraception reduces unplanned pregnancies (which contribute to population growth), and expansion of FP services would, therefore, serve as an effective lever against climate change.^50^ In response to this and similar arguments, other leading voices say that climate adaptation policies aimed at lowering population growth through FP are wrong, that population growth is not a main driver of climate change, and that the people and communities who have contributed the least to climate change and who bear the brunt of the impact of climate change (mostly in the Global South) would unjustly be the target of such policies.^51^^,^^52^

If one rightfully defines FP more broadly than contraceptive use—as a programming and policy framework “focused on helping individuals achieve their fertility intentions, which includes not only pregnancy prevention but also pregnancy planning, infertility counseling, SRH education, body literacy, and more,”^9^ and as a key component of SRH care^53^—then FP can and should be considered as a key component of climate adaptation, resilience action, and climate justice.^54^ In this broader definition, it is important to also consider emerging evidence in another direction: about the adverse impacts of air pollution, wildfires, heat stress, floods, and toxic chemicals on male and female fertility, the developing fetus, and obstetric outcomes^55^ and the relationship between environmental stressors and sexually transmitted infections, GBV, menstrual health and hygiene, and more.^25^ Newer efforts recognize these complex linkages and advocate for increased investments in dedicating climate adaptation financing that include girls’ education and modern voluntary FP as part of multisectoral climate adaptation approaches to ensure that those most vulnerable to climate change and its impacts have access to services in support of their basic human rights.^56^ A 2021 review of evidence found that there is a significant overlap between populations who have an increased vulnerability to climate change and populations who face socioeconomic, cultural, and political barriers to the realization of their SRH,^57^ further signaling the important role FP can play within a spectrum of interventions.

If one rightfully defines FP more broadly than contraceptive use, then FP can and should be considered as a key component of climate adaptation, resilience action, and climate justice.

### Prosperity

A wealth of FP-related research has focused on linkages with economic growth and employment topics of the SDG “prosperity” theme. This body of research has documented that the linkages vary according to differing contexts of work, gender norms, and structural inequalities.^48^ The “demographic dividend” is perhaps the most well-known example of FP being promoted as a prosperity “best buy” when included as part of a suite of investments, including investments in girls’ education and employment opportunities for youth.^58^^,^^59^ Critics have contended that the embrace of the demographic dividend by a wide range of development institutions poses a challenge to rights-based approaches to SRH and that enthusiasm for economic growth by accelerating the demographic transition through the rapid and wide-scale uptake of contraceptives marks a decisive step away from ICPD’s emphasis on individual rights and autonomy.^60^ A recent review on the impact of the demographic dividend at the country level in sub-Saharan Africa found that focus on the demographic dividend does not result in an outsized emphasis on FP. Rather, most sub-Saharan African countries have prioritized job creation and employment for youth, prompting calls for complementary investments in governance, FP, maternal and child health, education, and women’s empowerment.^61^ Pre-dividend countries (based on total fertility rate and projected share of working age population), such as Nigeria and Tanzania, have embraced multisectoral and integrated approaches to economic development working across health, education, labor, and gender equity.^62^ Given that many African countries themselves have prioritized investing in youth via improving education, expanding formal employment, and more—tenets that are front and center in the African Union’s Agenda 2063^63^—the demographic dividend remains a valuable advocacy and policy tool to attract investment in development.

### Peace

The SDG theme of “peace” champions the promotion of peaceful, just, and inclusive societies. Recent FP-related research has highlighted and strengthened the connections with the “peace” theme. For example, from a security standpoint, political demographers have found links between youthful age structures and increased risk of conflict.^64^ In response, they have urged countries to invest in programs that improve access to and quality of SRH services, efforts that promote girls’ educational attainment, and activities that advance women’s autonomy and rights as levers for peace and prosperity.^65^ This rationale for investing in SRH to advance peace is reinforced with evidence on the importance of investing in SRH to advance just and inclusive societies.

A focus on equity and inclusion has become central to advancing SRH policies and programming. Of course, to address inequities, it is essential to know where they exist, including among population subgroups, their geographical location, type of migration (if applicable), and more. Typically, wealth status has been used as a key identifier of these inequities.^66^ A more holistic approach is now recommended, guided in part by the USAID-supported High Impact Practices partnership, which suggests using data from the DHS and other population-based surveys to identify inequities across dimensions of availability, accessibility, and quality by disaggregating by age, education, marital status, wealth, residence, religion, and/or ethnic status.^67^ Recent work extended the framing of equity to include disability status, as well as sexual and gender minority status,^68^ with recognition that more work is required to ensure that FP programs are meeting the needs of everyone.^69^ Here, we intentionally use the word “everyone,” as it would be careless to only mention FP in the context of women and girls. Male engagement in FP, as supportive partners and contraceptive users, is a key component of gender transformative FP programming that “recognizes gender norms and inequalities, challenges and addresses them, and seeks solutions to overcome them.”^70^ Further, in recent years, global recognition of the importance of expanding access to people belonging to sexual and gender minority groups has grown exponentially, as has advocacy for FP programs to address the needs of lesbian, gay, bisexual, transgender, queer, and intersex populations.^71^ Equity for these populations requires good foundational data, and the USAID-supported DHS Program is currently exploring how to safely and in a standardized fashion, include sexual and gender minority-related topics in surveys, building, in part, from lessons learned when DHS worked with the Washington Group on Disability Statistics to develop the disability questionnaire module in 2016.^72^ The DHS Program is also exploring new ways of capturing GBV in surveys, including emerging routes of technology-facilitated GBV. These are small examples of big steps USAID is taking to understand and, thus, design interventions for what the global community lumps together as “underserved and marginalized communities.” If the adage “what gets measured gets done” has any relevance to the ICPD Programme of Action, it’s this aspect of shining a light on who is unmeasured, and understanding who these communities are in all their diversity to truly create a more inclusive future. Indeed, there is great potential in harnessing technology to reach or amplify outreach to groups that may be missed in traditional surveys or population registers. This includes adolescents and youth, who may be more likely to engage with FP and health messaging via video games and direct-to-consumer approaches or by using convenient self-care FP methods.^73^

### Partnership

Finally, we examine FP’s role toward advancing the “partnership” SDG theme. In recent years, a global consensus has been affirmed and reaffirmed, including in a donor statement penned by USAID and 3 dozen bilateral donors and foundations, that success for all donor-supported programs (not just FP) hinges upon intentional collaboration and cooperation between donors and the people, institutions, and communities who address and are impacted by these challenges every day.^74^ USAID and other donors are embracing localization strategies, which include shifting power, directly channeling funding to local actors, and more.^21^ Whether partnership takes the form of costed implementation plans developed through a strategic government-led consultative process that results in a detailed plan for program activities and an estimate of the funding required to achieve an established set of goals^75^ or a more comprehensive approach that includes capacity-strengthening, social behavior change, or data demand,^76^ donors must remain agile to respond to country needs. This is sometimes easier said than done, given stagnant or dwindling budgets and shifting donor priorities, which may lead to unpredictability disruptive to partner country planning and implementation, as revealed in a recent analysis of aid in Kenya, Tanzania, Uganda, and Zambia.^77^ Despite these challenges, partnership is and must continue to be the cornerstone of FP assistance programs. Indeed, global partnerships that engage multilaterals, private organizations, and governments alike, such as FP2030 and the Ouagadougou Partnership, have been and remain key to accelerating country-led FP goals. ^78^

A global consensus has been reaffirmed that success for all donor-supported programs (not just FP) hinges upon intentional collaboration and cooperation between donors and the people, institutions, and communities who address and are impacted by these challenges every day.

## LOOKING TO THE FUTURE

Since the 25th anniversary of ICPD, USAID has made great strides in accelerating its commitment to localization while ensuring no one is left behind.^21^ As we have illustrated, FP is a necessary component of sustainable development. USAID’s ongoing support for this important contribution to SRH remains steadfast. Given the state of research and discussions we have outlined and recognizing that the ICPD Programme of Action remains an enduring vision and approach with goals yet to be achieved, USAID continues to uphold the principles of agency, quality, equity, and inclusion into its SRH and development programs. Recognizing that advancing the Programme of Action requires interventions within and beyond FP,^79^^,^^80^ USAID’s Primary Impact initiative accelerates progress in health and survival through investment in primary health care,^81^ a key step in reducing maternal mortality. In collaboration with partners around the world, USAID is also contributing to redefining “success” and how it is measured and is continually exploring how best to meet the varied and expanding SRH needs in our dynamic world. Take, for instance, several recent examples from Zambia, Nigeria, and Ethiopia.

In Zambia, vague policies on age of consent for FP services limit adolescents’ access to and use of FP services, which contributes to high adolescent pregnancy rates, high maternal and child mortality rates, and constrained educational and economic opportunities for first-time parents.^82^ USAID is working with the Government of Zambia and local partners to leverage Zambia’s otherwise strong FP program, including its sound commodity security plan, to address adolescent SRH needs. This includes taking a total market approach to expand access to contraceptives and FP services via the private sector and partnering with local stakeholders to encourage policy reform.

In Nigeria, USAID works closely with the government-led National RH Technical Working Group to advance an evidence-based expansion of the contraceptive method mix. USAID, alongside other donors, supported the Government of Nigeria to introduce and scale up new and underutilized methods to broaden the range of contraceptive choices. Similarly, in alignment with the global FP community’s shift to person-centered, rights-based programs that promote individual and collective reproductive empowerment and agency, the Government of Nigeria (in one of the first instances globally of adapting the World Health Organization guidelines), with USAID support, issued national guidelines on self-care for sexual, reproductive, and maternal health,^83^ including a focus on FP self-care. USAID/Nigeria plans continued support for the contextualized implementation of FP High Impact Practices, especially those that integrate a full range of FP methods into antenatal, labor and delivery, and postpartum care and expand access to FP self-care.

In Ethiopia, innovative financing approaches have helped the country make progress in FP outcomes, with learnings for other health areas. Following the Ministry of Health’s call for support to address funding gaps for FP commodity procurement, USAID/Ethiopia collaborated with the Ministry of Health, other development partners, and key stakeholders to increase domestic financing for FP and RH commodity procurement and for improved quality of FP services. This collaborative effort led to the signing of a compact agreement between the Government of Ethiopia and other development partners to co-finance FP commodity procurement through a matching fund from the Government’s treasury with a total amount of about US$36 million for 3 years (2023–2025). The Government of Ethiopia’s commitment to increasingly co-finance FP commodity procurement came when the country’s fiscal space was highly constrained and reserve funds were hugely depleted due to the conflict in Northern Ethiopia. The Government has committed financing of 25%, 50%, and 85% of total contributions for the first 3 years, respectively. This is a big step toward the realization of the country’s FP2030 commitment. The government of Ethiopia now recognizes this cost-share approach as a promising funding arrangement for other health programs.

Looking to the future, USAID’s *Pathways to Progress for Sexual and Reproductive Health*, *2024–2030*^84^ builds on long-standing commitments. It outlines the direction of USAID’s FP/RH program through 2030 by articulating a clear destination—a world where ongoing improvements to SRH contribute to longer, healthier, and more prosperous lives for all—and detailing evidence-based pathways to reach that destination.^2^ The 3 identified pathways operate at individual, systems, and societal levels.

- Individuals have accurate information, skills, and ability to take action to achieve the highest attainable levels of SRH across their lifetime.
- Health systems provide quality, accessible, and people-centered SRH care.
- Local communities, organizations, institutions, and governments create and foster social norms and policies that support individuals to make and act on their own SRH decisions, free from violence, coercion, and discrimination.

Through the principles and strategies outlined in Pathways, USAID commits to recalibrating its FP/RH program to accelerate progress and address gaps. This evolution is grounded in evidence-based strategies focused on meeting individuals’ SRH needs by championing reproductive agency, strengthening quality health systems, and fostering supportive social norms and policies. By enacting these commitments and strengthening collaboration with partner countries and donors alike, USAID can help shape a world where ongoing improvements to SRH contribute to longer, healthier, and more prosperous lives for all. These collective efforts will garner transformational benefits to women, families, communities, and countries by accelerating progress across the 5 SDG themes of people, planet, prosperity, peace, and partnership while bringing us closer to the bold, people-centered vision described 3 decades ago in the ICPD Programme of Action.

## Notes

**Peer Reviewed**

**First Published Online:** October 11, 2024.
